# Developing the optimal gross movement interventions to improve the physical fitness of 3–10 year-old children: a systematic review and meta-analysis

**DOI:** 10.3389/fpsyg.2024.1355821

**Published:** 2024-10-01

**Authors:** Li Hui, Sun Wei, Qu Luping, Guo Nannan

**Affiliations:** ^1^Department of Physical Education, Qufu Normal University, Qufu, Shandong, China; ^2^Department of Sports Training, Tianjin University of Sport, Tianjin, China; ^3^Department of Physical Education, Donghu Primary School, Tianjin, China

**Keywords:** gross movement, children, physical fitness, meta-analysis, 3–10 years old

## Abstract

**Objective:**

To identify the optimal parameters of gross movement interventions to yield the strongest effects on physical fitness among children aged 3–10 years and to provide a reference for the development of gross movement interventions to improve the physical fitness of children.

**Background:**

There has been a global decline in children’s physical fitness. Previous studies have shown that gross movement interventions can improve children’s physical fitness, but the optimal intervention parameters for achieving the strongest effects have yet to be determined. Therefore, we conducted this meta-analysis to determine the optimal intervention parameters for yielding the strongest effects on children’s physical fitness.

**Methods:**

We searched the Web of Science, PubMed, China Biology Medicine, China National Knowledge Infrastructure, Wanfang Data, and China Science and Technology Journal databases to identify randomized controlled trials on the effects of gross movement interventions (walking, running, jumping, throwing, batting, rolling, spinning, catching, and leg lifting) on children’s physical fitness (upper-body strength, lower-body strength, explosive power, speed, flexibility, and balance). We included studies published up to September 2023. The inclusion and exclusion criteria were developed based on the PICOS framework, and the quality of the included studies was evaluated. Subgroup analysis was performed using Review Manager 5.3, and the data were pooled using a random effects model to obtain the SMD (or WMD) and 95% confidence intervals (CIs).

**Results:**

A total of 23 studies involving 2007 healthy children aged 3–10 years met the inclusion criteria. Gross movement interventions significantly improved the explosive power of children aged 3–10 years [WMD, 6.2]. The most effective intervention duration was 16–18 weeks [WMD, 0.45]. The most effective intervention frequency was one session per week [WMD, 1.06]. The optimal duration of single sessions was 60 min [WMD, 0.47]. Children aged 7–10 years [WMD, 1.41] showed the most significant improvements in physical fitness after gross movement interventions.

**Conclusion:**

Gross movement interventions had a positive effect on the physical fitness of children aged 3–10 years. The optimal intervention parameters include 60-min sessions once a week across a total duration of 16–18 weeks.

## 1 Introduction

Physical fitness is a term that comprehensively describes physical performance related to human structure and function (Chen et al., 1993) and includes the following qualities: speed, endurance, strength, coordination, flexibility, and balance (He, 2017; Tao et al., 2017; Kim et al., 2021; Yang et al., 2022). In this study, upper-body strength, lower-body strength, explosive power, speed, flexibility and balance were included as dependent variables. Many studies have confirmed the relationship between children’s physical fitness and health. Some studies have shown that improving children’s physical fitness can prevent or reduce the risk of obesity and cardiovascular disease (Al-Mallah et al., 2018; Elagizi et al., 2018; Henriksson et al., 2020). Improving children’s physical fitness can also lead to long-term health benefits (Smith et al., 2014; Mintjens et al., 2018). However, there has been a global decline in children’s physical fitness (Burns et al., 2018; Godoy-Cumillaf et al., 2021; Qu, 2021). According to a new report jointly released by the World Health Organization and the United Nations Children’s Fund, there are approximately 317 million cases of physical development disorders among children worldwide (World Health Organization, 2023). In 2022, China issued the Fifth National Physical Fitness Monitoring Communique, and compared with 2014, many physical qualities of Chinese children have shown a downward trend—for example, strength and flexibility decreased by 1.3–6.6% (National Health Monitoring Centre, 2022). In developed countries, the strength of children continues to decline (Tomkinson, 2007), as exemplified by the decreases in muscular strength among children in Sweden and Russia (Malina and Katzmarzyk, 2006) and decreases in upper-body strength (Tremblay et al., 2010) and lower-body strength (Moliner-Urdiales et al., 2010) among Canadian and Spanish children.

Physical fitness and gross movement are the two main aspects of physical performance (Huang, 2000) and are measured from different perspectives to explain physical performance. Physical fitness is a stable external feature of physical performance (Zhou, 2010), while gross movement is the basic foundation of physical performance (Li et al., 2019) and includes the earliest basic motor skills established by children (Wang et al., 2020). Gross movements, which are completed using large skeletal muscle groups (Thomas et al., 2022), mainly include basic motor abilities (e.g., walking, running, jumping, throwing, catching, hitting, kicking, and batting). In this study, walking, running, jumping, throwing, batting, rolling, rotating, catching, and leg lifting were included as independent variables. Gross movement plays a significant role in improving children’s physical fitness (Wu et al., 2014; Zhou, 2020) and reducing the risk of obesity and cardiovascular and other health problems in children; therefore, gross movement interventions have become an important parameter for studying children’s health promotion in the fields of sports and epidemiology (Okely et al., 2004; Barnett et al., 2008; Robinson et al., 2015; Ma and Song, 2017; Pu and Yang, 2017; Hao, 2018; Li et al., 2019; Wang et al., 2019). Additionally, in recent years, most related studies have focused on examining the correlation between gross movement performance and physical fitness (Foulkes et al., 2021; Liu et al., 2023) or differential performance (Haugen and Johansen, 2018; Zheng et al., 2022) in children, thus confirming the important impact of gross movement interventions on improving children’s physical fitness in early development. This positive correlation persists as age increases (Robinson et al., 2015), thus laying the foundation for future exercise ability and fitness levels. Therefore, it is particularly important to consider gross motor development in childhood. However, the optimal parameters for gross movement interventions to yield the greatest improvements in physical fitness among children aged 3–10 years remain unclear.

Three-year-old children perform the most basic physical activities, such as walking and climbing (Xu et al., 2023); they can have the potential to master gross movements at age 6 (O’Brien et al., 2016)and are expected to reach proficiency at age 10 (Ulrich, 2000). However, according to statistics, the gross movement level of 10-year-old children in Germany, China, Brazil and other countries is not ideal. The gross movement level of 10-year-old children worldwide has failed to match the standards for their age group (Xu et al., 2023). Therefore, this study quantified the beneficial effects of gross movement interventions on children’s physical fitness, providing theoretical support for further research in this field.

Many countries have adopted the standing long jump, tennis ball long throw, two-foot continuous jumping, 10-meter shuttle run, balance beam walking, and sit-and-reach tests for evaluating children’s physical fitness in terms of strength, speed, agility and flexibility (State General Administration of Sport, 2015; Wang, 2022). Although gross movement interventions can effectively improve children’s physical fitness, it is still necessary to identify more accurate strategies for children aged 3–10 years to carry out accurate and effective interventions and to fully prepare children for future healthy development. Previous studies have shown that age, intervention duration, and intervention frequency may be significant factors associated with the effect of gross movement interventions on children’s physical fitness (Pozuelo-Carrascosa et al., 2018; Utesch et al., 2019; Godoy-Cumillaf et al., 2020; Wu et al., 2021). Therefore, it is very important to accurately identify the optimal parameters for gross movement interventions to yield the greatest improvements in physical fitness among children aged 3–10 years.

Therefore, the purpose of this study was to explore the optimal parameters for gross movement interventions to yield the greatest improvements in physical fitness among children aged 3–10 years and to provide a reference for the development of gross movement intervention programs that aim to improve the physical fitness of children.

## 2 Methods

### 2.1 Retrieval strategy

Two researchers conducted a literature search with the following primary search terms: “Physical Fitness AND gross movement AND randomized controlled trial.” Additionally, the following secondary search terms were used: “Fitness, Physical OR Physical AND gross action OR gross motor skill OR large muscle action OR basic motor skills OR functional training OR functional game OR functional sports game OR basic movement skills OR function training AND randomized OR toddler OR Child OR childhood OR baby OR primary school OR Kindergarten.” The following databases were searched: Web of Science, PubMed, China Biology Medicine, China National Knowledge Infrastructure, Wanfang Data, and China Science and Technology Journal. We included studies from China, the United Kingdom and Mexico. The retrieval time ranged from the establishment of the database to September 2023. If the articles were incomplete or unavailable, we contacted the corresponding authors by email to obtain detailed information. The search initially yielded 3,680 studies on the effects of gross movement interventions on the physical fitness of healthy children aged 3–10 years.

### 2.2 Inclusion and exclusion criteria

Two researchers independently screened the articles, and disagreements were resolved by consulting a third researcher. The inclusion criteria were developed in accordance with the population, intervention, comparison, outcomes, study design (PICOS) framework. (1) Study subjects: All the subjects were healthy children aged 3–10 years without disease. (2) Intervention measures: The experimental group performed intervention exercises involving gross movements (walking, running, jumping, throwing, batting, rolling, spinning, catching, and leg lifting). (3) Control measures: The control group participated in normal kindergarten or primary school activities without additional intervention. (4) Outcome indicators: All or some of the six outcome indicators, including a 10-m shuttle run, standing long jump, tennis ball long throw, sit-and-reach, two-foot continuous jumping, and balance beam, were included. The outcome data are expressed as the mean ± SD. (5) Study type: The trial design was a randomized controlled trial. The exclusion criteria were as follows: (1) descriptive research, analytical research, conference abstracts and literature reviews; (2) duplicate studies, studies of low quality, and studies of which the full text was not available; (3) studies that did not report the outcomes as the mean ± SD and for which the data could not be converted. The measurement evaluation criteria were scored.

### 2.3 Data extraction

Two researchers independently extracted the following data according to a predefined protocol: literature name, author, publication time, nationality of subjects, sex, age, total number of subjects, number of experimental groups, number of control groups, intervention measures, intervention duration, single intervention duration, and outcome indicators. For the outcome indicators, the physical fitness test scores before and after each RCT in the experimental group and the control group were recorded. Scores are reported as the mean ± SD. Disagreements were resolved by consulting a third investigator.

### 2.4 Method quality evaluation

The Cochrane risk of bias tool was used to evaluate the quality of the 23 included studies across seven domains: random allocation method, concealment of allocation scheme, blinding of participants and implementers, blinding of outcome evaluators, integrity of outcome data, selective report results, and other sources of bias. Each domain was rated as “high risk,” “unclear risk” or “low risk.” Two researchers independently assessed the data. Any disagreements were resolved by consulting a third researcher.

### 2.5 Statistical analysis

STATA 16.0 was used for the statistical analysis. Since the indicators included in the literature were continuous outcome variables, a random-effects model was used to conduct a meta-analysis based on 6 outcome indicators (sit-and-reach, standing long jump, 10-m shuttle run, tennis ball throw distance, two-foot continuous jumping, balance beam), combined with effect size (WMD or SMD) and 95% confidence intervals. The weighted mean difference (WMD) eliminates the influence of absolute values on the results so that the research results can truly reflect the experimental effect; in addition, it is easy to understand when applied. The standardized mean difference (SMD) not only eliminates the effect of absolute values but also eliminates the effect of weights and measures on the results. The larger the absolute value is, the more obvious the effect. The I^2^ test and Q test were used to evaluate the heterogeneity of each index. When *P* ≥ 0.1 and I^2^ ≤ 50%, there was homogeneity or low heterogeneity among the studies; in such cases, a fixed-effects model was used for analysis. When *P* < 0.1 and I^2^ > 50%, there was strong heterogeneity between studies; in such cases, a random-effects model was used for analysis. Subgroup analysis was conducted to calculate the influence of gross movement interventions on children’s physical fitness based on the following factors: different physical fitness indicators, age, intervention duration, intervention frequency and single intervention duration.

## 3 Results

### 3.1 Literature search results

**FIGURE 1:**
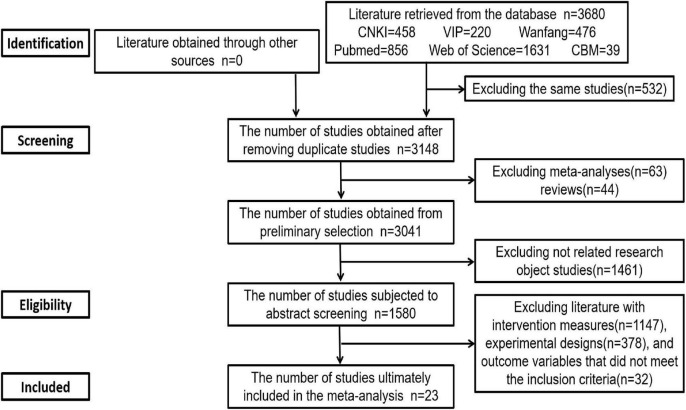
Flow chart of the literature retrieval and study selection process.

The results of the literature search and the study selection process are shown in Figure 1. All literature screenings were performed independently and in a double-blinded manner by 2 researchers (L.H. and S.W.) based on the inclusion and exclusion criteria. The search initially yielded 3,680 articles. First, the literature was classified, and duplicate studies were eliminated, leaving 3,148 studies. Second, after excluding 107 studies that were meta-analyses, reviews, or systematic reviews and 1,461 studies that were not related to the current subject, 1,580 studies remained. Then, 1,557 articles were excluded after full-text screening due to the intervention, trial design, or outcome variables not meeting our inclusion criteria. Finally, 23 studies were included in the meta-analysis. After screening, the two researchers compared the selected studies (Kappa≈0.65); therefore, the selected literature was considered reliable, and any disagreements were resolved by discussion with a third researcher (Q.L.P.).

### 3.2 Study characteristics

The main characteristics of the included studies are shown in Table 1. A total of 2,007 healthy children aged 3–10 years were included in the studies (1,037 in the experimental group and 970 in the control group). Nine studies included six outcome measures. The children’s ages ranged from 3 to 10 years. The intervention duration ranged from 4 to 36 weeks. The intervention frequency ranged from 1 to 3 times per week. The duration of single sessions ranged from 30 to 60 min. Subgroup analysis was performed based on the following factors: physical quality factors (flexibility, explosive power, speed, upper-body strength, lower-body strength, balance); age (3–6 years old, 7–10 years old); duration of intervention (4–8, 10–12, 16–18, and 36 weeks); intervention frequency (1 time/week, 2 times/week, 3 times/week); and duration of a single session (30, 40, 45, and 60 min).

**TABLE 1:** Basic characteristics of the included studies.

References	Nationality	Male: Female	Age (year)	Sample size	Experimental group sample size	Control group sample size	Intervention measure	Intervention frequency (times/week)	Duration of intervention (weeks)	Duration of a single session (minutes/session)	Primary outcome measure
Zou, 2016	China	42:42	10	84	42	42	Run, walk, pull, crawl, toss	3	16	40	①
Liu, 2014	China	53:34	5–6	87	45	42	Run, crawl, stand, roll, jump	3	12	40	①②③④⑤⑥
Wu, 2015	China	21:19	5–6	40	20	20	Run, jump, stand, pull	3	12	60	①②③④⑤⑥
Liu, 2016	China	/	9–10	60	30	30	Leg lifts, stretches, jumps, Throw the ball, run, crawl	3	12	/	①
Wright et al., 2015	United Kingdom	/	10	22	11	11	Run, crawl, squat, walk	2	4	/	①
Wei and Wei, 2016	China	12:8	9–10	20	10	10	Walk, squat, rotate, and lift your legs	2	8	/	①②
Liu, 2018	China	/	5	129	65	64	Run, jump, bounce the ball, catch the ball, slide forward, slide sideways, kick the ball, roll the ball	2	16	45	①②③④⑤⑥
Wang et al., 2018	China	54:0	9–10	40	20	20	Walk, run, stretch, dribble, shoot, pass	3	10	60	①②
Huang and Gao, 2018	China	/	9–10	72	36	36	Functional physical training (including gross movements)	3	16	/	①
Wang, 2020	China	/	9–10	54	28	26	Squat, high leg lift, jump, lunges, spins, major muscle stretches, ball lifts	3	12	/	①
Du et al., 2019	China	116:126	10	242	125	117	Running, jumping, ball games, roll, throw	1	18	/	①
Xia, 2020	China	/	4–5	61	21	18	Climb, stand, throw, walk, dribble, shoot, balance, jump	1/2	12	40	①②③
Zhao, 2020	China	31:29	5	60	30	30	Dribble, crawl, sidestep, throw, roll, set, toss, catch	3	16	40	①②③④⑤⑥
Jaksic et al., 2020	Mexico	72:60	4–7	132	66	66	Jumping, running, tumbling, throwing, ball games	2	36	60	①②
Gao, 2021	China	/	3–6	112	59	53	Catch the ball, dribble, kick the ball, throw the ball, jump, bounce the ball, step forward, step sideways	2	12	30–40	①②④⑤⑥
Xu, 2021	China	64:67	4–5	131	67	64	Functional play, stability skills, mobility skills, maneuverability skills, physical fitness activities	3	12	/	②③④⑤
Wang, 2021	China	/	4–5	104	54	50	Walk, climb, run, jump, throw, catch, kick, bounce, roll, dribble, stand	3	16	30	①②④⑤⑥
Zhou et al., 2021	China	/	4–5	173	94	79	Body motor function training methods (including gross movements)	3	18	40	①②③④⑤⑥
Chen, 2022	China	40:40	4–5	80	40	40	Toss, throw, bounce, kick, run, jump, slide, stretch, spin, shake, balance	3	10	45	①②③④⑤⑥
Ding, 2021	China	20:20	4–6	40	20	20	Run, dribble, catch, chase, toss	1	12	40	①②③④⑤⑥
Zheng, 2022	China	/	3–6	60	30	30	Run, jump, throw, bounce, kick, catch, roll	2	8	30	①②④⑤⑥
Zhou, 2022	China	60:60	4–5	120	60	60	Walk, run, jump, slide, crawl, balance, throw, catch, kick, bounce	3	12	40	①②③④⑤⑥
Fan, 2022	China	52:32	4–5	84	42	42	Functional sports games (including gross action)	3	12	45	①②③④⑤⑥

“①” means the sit-and-reach test, “②” means the standing long jump, “③” means the 10-meter shuttle run, “④” means the tennis ball throw for distance, “⑤” means two-foot continuous jumping, and “⑥” means the balance beam.

### 3.3 Risk of bias

**FIGURE 2:**
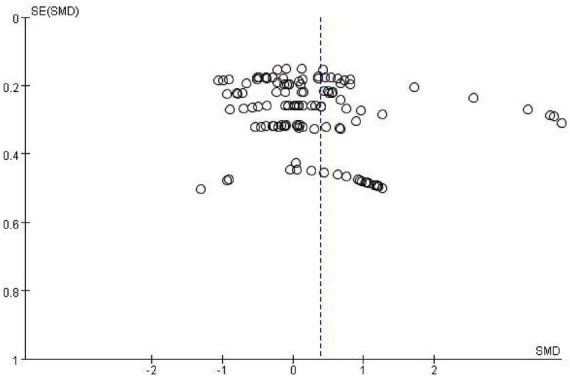
Funnel plot.

**FIGURE 3:**
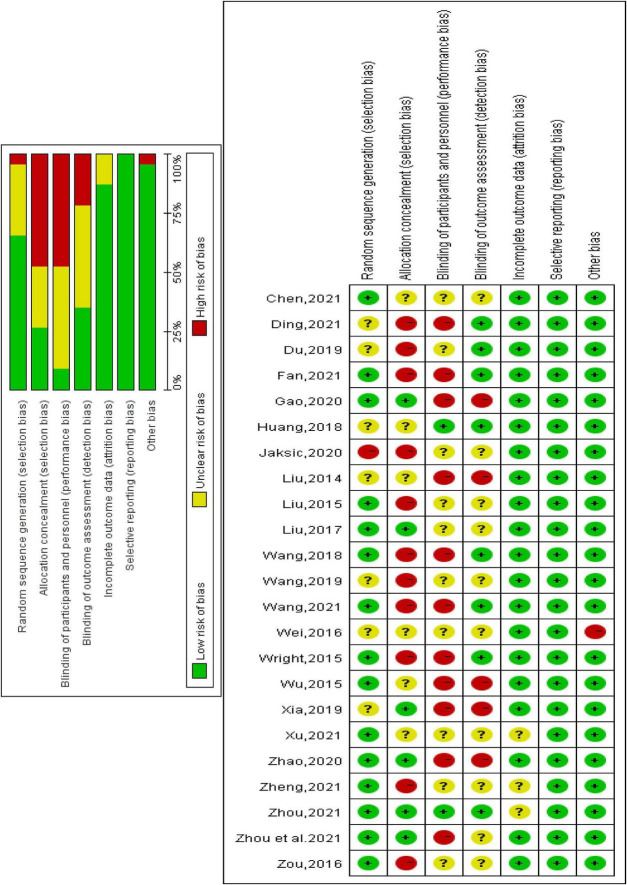
Results from the Cochrane risk of bias tool.

The Cochrane risk of bias tool was used to evaluate the methodological quality of the included studies across six domains: selection bias, implementation bias, detection bias, loss of access bias and reporting bias (Figure 2). The kappa of the two researchers was approximately 0.62, so the literature quality evaluation was reliable. The risk of bias for each domain was categorized as low, unclear, or high, as shown in Figure 3.

### 3.4 Results of the meta-analysis

#### 3.4.1 The influence of gross movement interventions on children’s physical fitness

**FIGURE 4:**
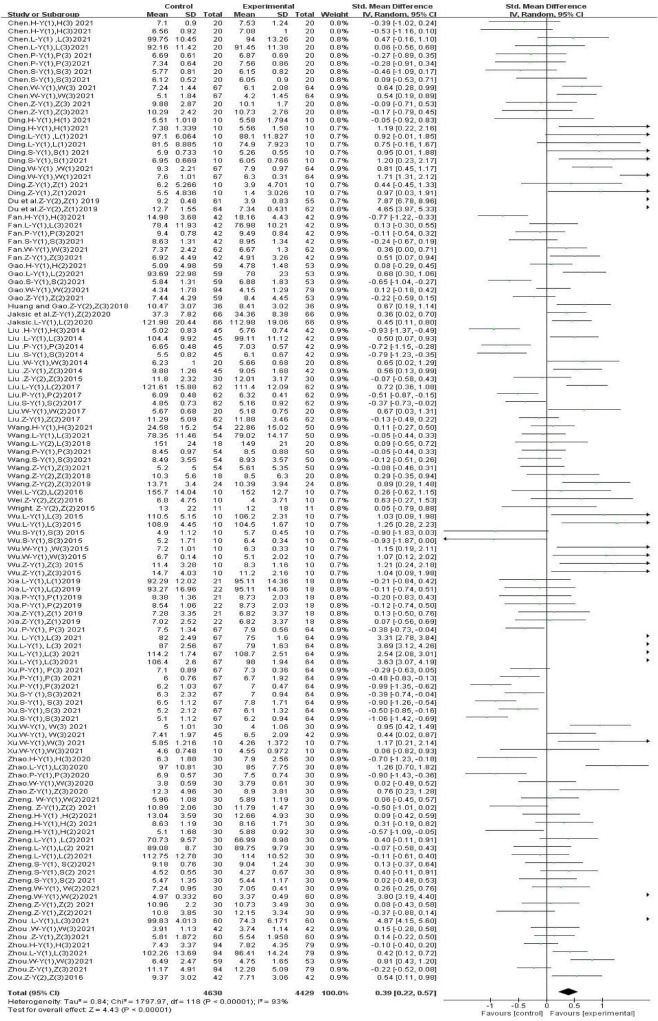
The effects of gross movement interventions on physical fitness.

After analyzing the data from all included studies, we found that gross movement interventions improved physical fitness in children aged 3–10 years [SMD, 0.39 (95% CI, 0.22–0.57); *P* < 0.05], and there was significant heterogeneity (I^2^ = 93%) (Figure 4). However, this effect did not differ according to various physical fitness factors, age, intervention duration, intervention frequency, or single-session duration. Therefore, a subgroup analysis was performed (Table 2).

**TABLE 2:** Summary of subgroup analysis results.

Grouping standard	Groups	Effect size
Physical fitness factors	Explosive power	6.20
	Flexibility	1.34
	Balance	-1.23
	Lower-body strength	-0.86
	Upper-body strength	0.79
	Speed	-0.72
Age range	3–6 years old	0.18
	7–10 years old	1.41
Intervention duration	4–8 weeks	0.03
	10–12 weeks	0.14
	16–18 weeks	0.45
	36 weeks	0.41
Intervention frequency	One time/week	1.06
	Two times/week	0.14
	Three times/week	0.13
Single-session duration	30 min	-0.01
	40 min	-0.05
	45 min	-0.12
	60 min	0.47

#### 3.4.2 Subgroup analysis: effects of gross movement interventions on different physical fitness factors in children

**FIGURE 5:**
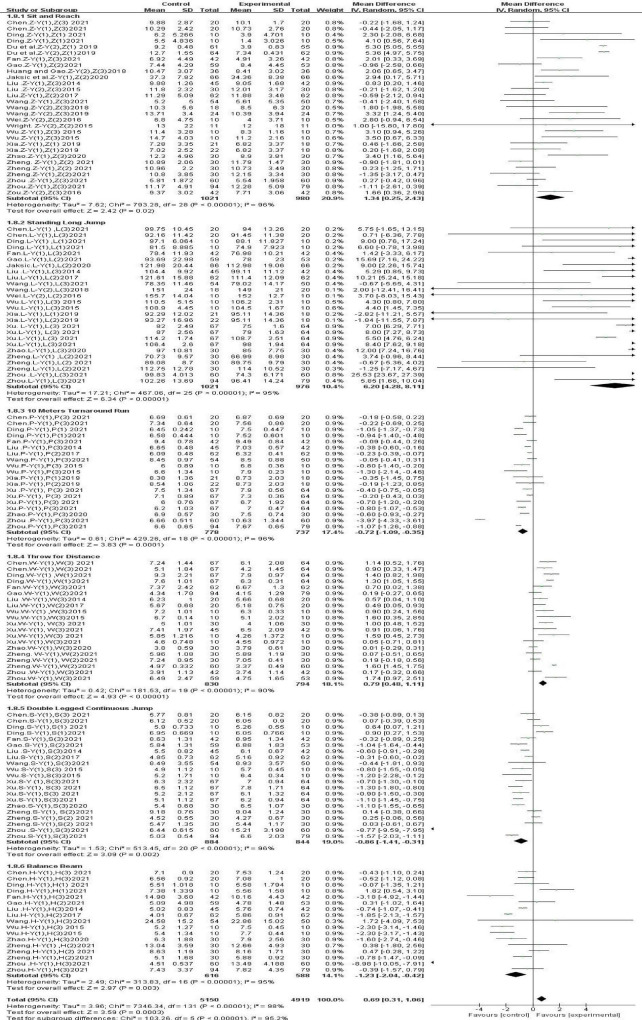
Different physical fitness factors.

The results varied when examining different physical fitness factors (Figure 5). Specifically, gross movement interventions significantly improved the following physical indicators: explosive power [WMD, 6.2 (95% CI, 4.28–8.11); *P* < 0.05], flexibility [WMD, 1.34 (95% CI, 0.25∼2.43); *P* < 0.05], balance [WMD, −1.23 (95% CI, 1.23∼0.42); *P* < 0.05], lower-body strength [WMD, 0.86 (95% CI, 1.41∼0.31); *P* < 0.05], upper-body strength [WMD, 0.79 (95% CI, 0.48∼1.11); *P* < 0.05], and speed [WMD, 0.72 (95% CI, 1.09∼0.35); *P* < 0.05]. However, there was significant heterogeneity in all six subgroups (I^2^ > 90.0%).

#### 3.4.3 Subgroup analysis: effects of gross movement interventions on the physical fitness of children of different ages

**FIGURE 6:**
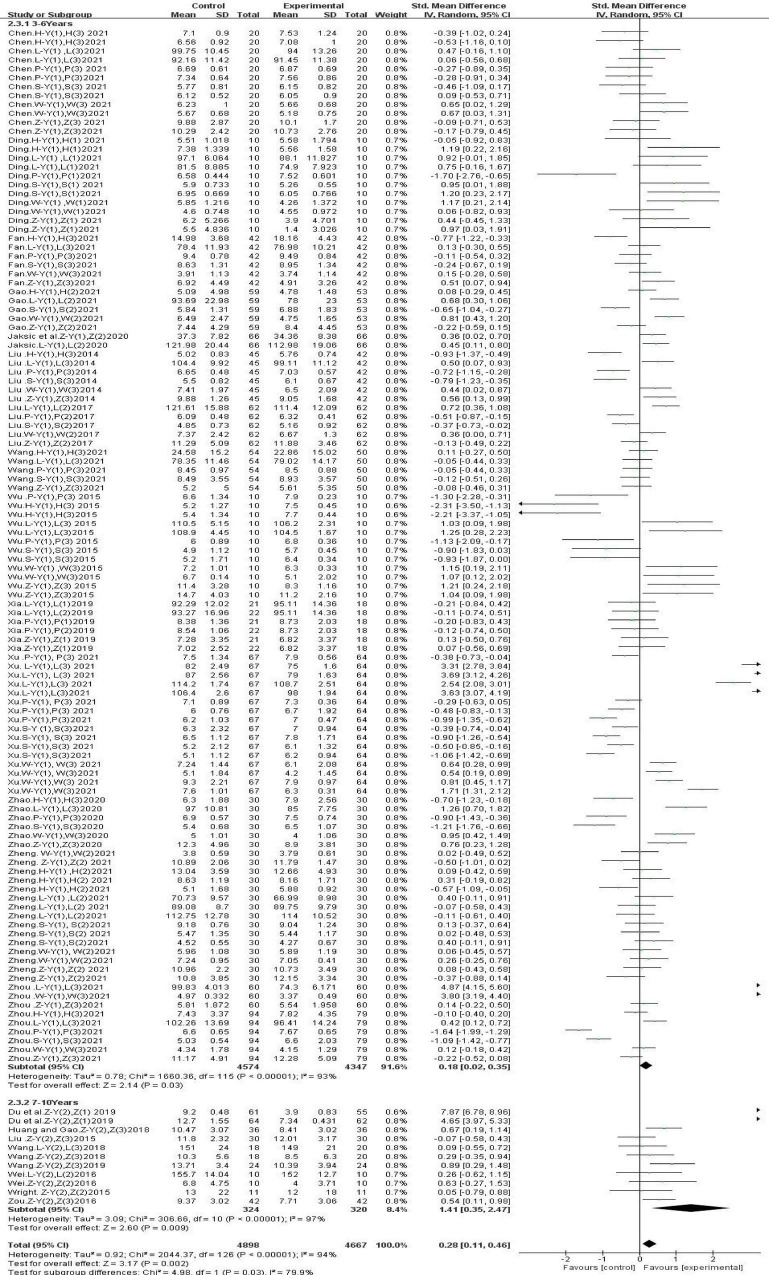
Different ages.

The analysis results (Figure 6) showed that gross movement interventions significantly improved the physical fitness of children aged 7–10 years [SMD, 1.41 (95% CI, 0.11–0.46); *P* < 0.05], and there was significant heterogeneity (I^2^ = 97.0%). There was a small beneficial effect of gross movement interventions on the physical fitness of children aged 3–6 years [SMD, 0.18 (95% CI, 0.02–0.35); *P* < 0.05], and there was significant heterogeneity (I^2^ = 93.0%).

#### 3.4.4 Subgroup analysis: the effect of different intervention durations on gross movement interventions on children’s physical fitness

**FIGURE 7:**
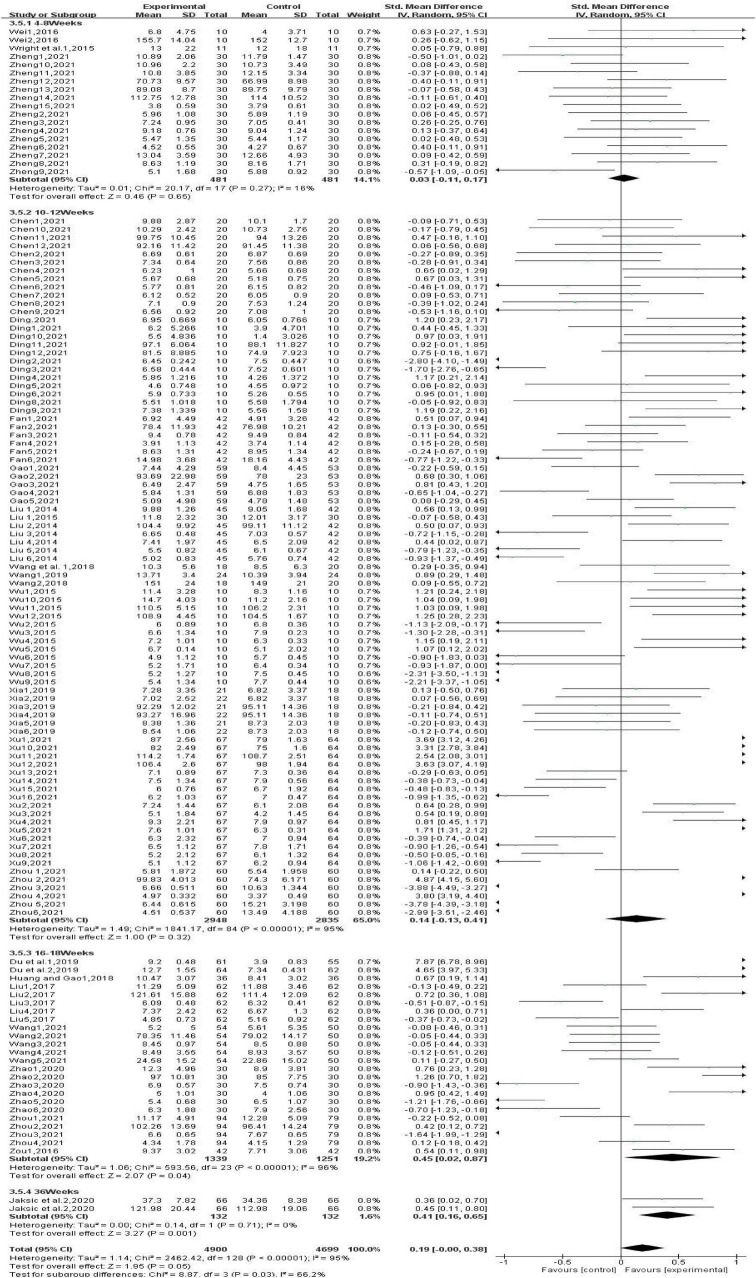
Different intervention durations.

Subgroup analysis based on different intervention durations showed (Figure 7) that a gross movement intervention lasting 4–8 weeks had no significant effect on children’s physical fitness [SMD, 0.03 (95% CI, −0.11 to 0.17); *P* > 0.05]. Gross movement interventions lasting 10–12 weeks had a small beneficial effect on children’s physical fitness [SMD, 0.14 (95% CI, −0.13 to 0.41); *P* < 0.05]. Gross movement interventions lasting 16–18 weeks significantly improved children’s physical fitness [SMD, 0.45 (95% CI, 0.02∼0.87); *P* < 0.05]. Gross movement interventions lasting 36 weeks also significantly improved children’s physical fitness [SMD, 0.41 (95% CI, 0.16–0.65);*P* < 0.05].

#### 3.4.5 Subgroup analysis: effects of gross movement interventions at different intervention frequencies on children’s physical fitness

**FIGURE 8:**
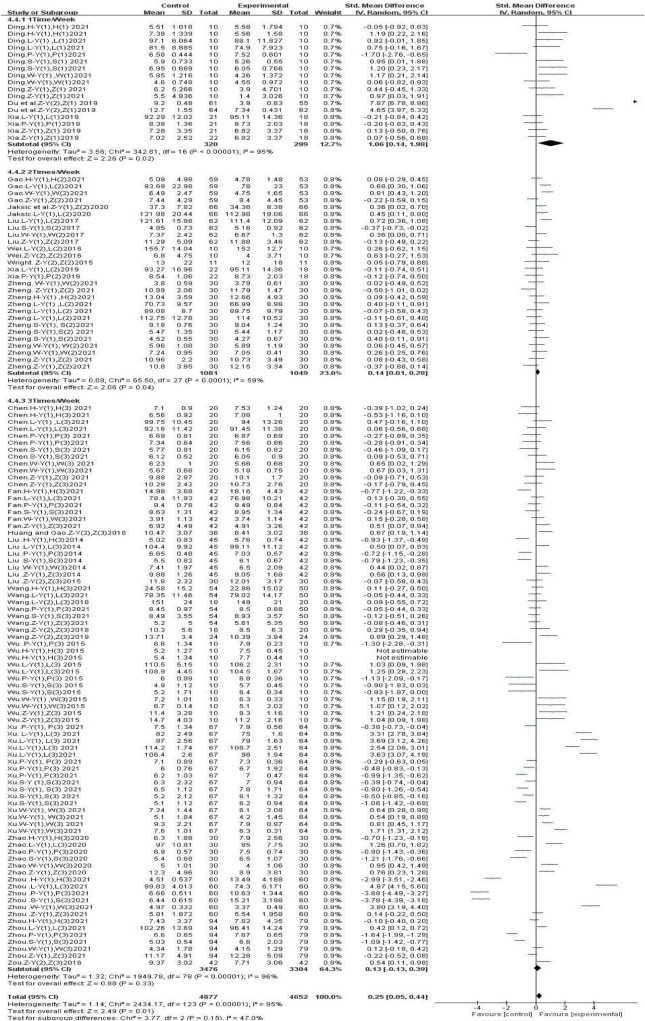
Different intervention frequencies.

Subgroup analysis based on intervention frequencies showed (Figure 8) that one gross movement intervention per week significantly improved children’s physical fitness [SMD, 1.06 (95% CI, 0.14–1.98); *P* < 0.05]. Two [SMD, 0.14 (95% CI, 0.01∼0.28); *P* < 0.05] and three [SMD, 0.13 (95% CI, −0.13∼0.39); *P* < 0.05] gross movement interventions per week had small beneficial effects on children’s physical fitness. However, there was significant heterogeneity (I^2^ > 90.0%).

#### 3.4.6 Subgroup analysis: the effect of different single-session durations of gross movement interventions on children’s physical fitness

**FIGURE 9:**
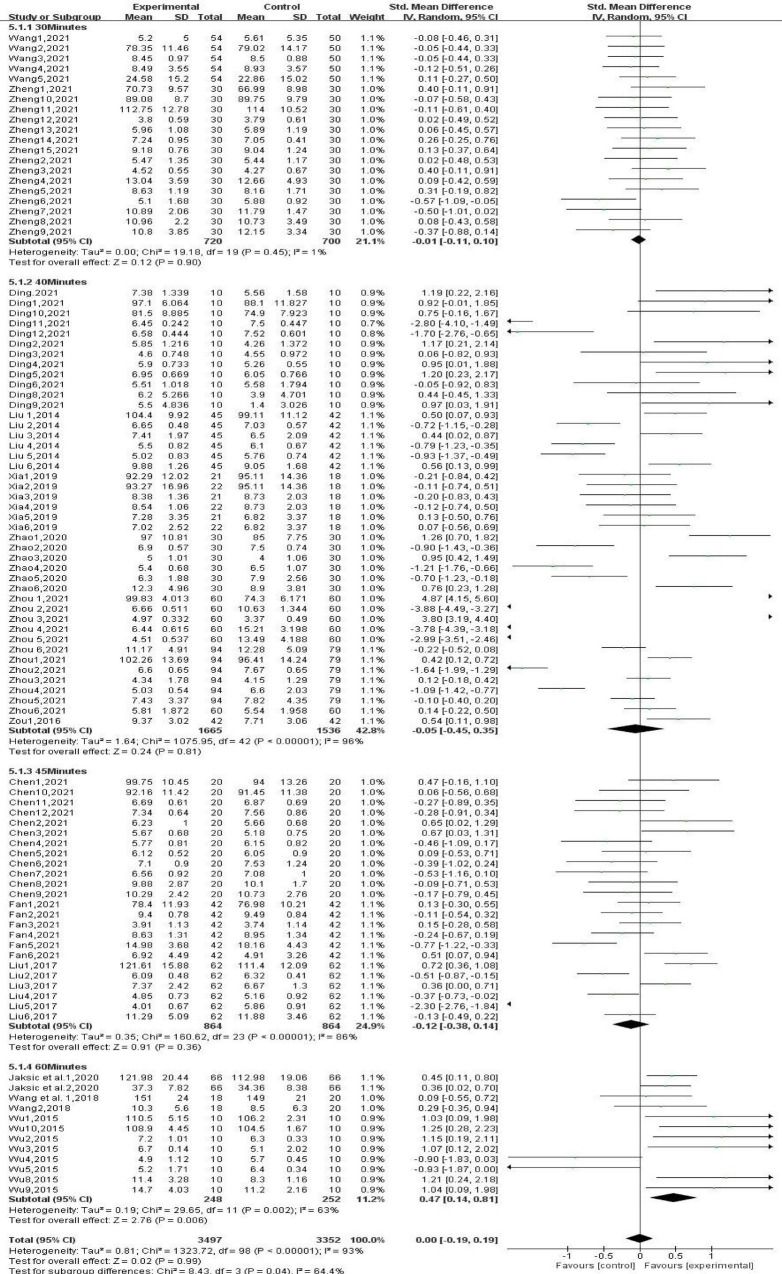
Different single-session durations.

Subgroup analysis based on single-session durations (Figure 9) showed that 30-min gross movement interventions did not have a significant effect on children’s physical fitness [SMD, −0.01 (95% CI, −0.11∼0.10); *P* > 0.05]. Furthermore, 40-min [SMD, −0.05 (95% CI, −0.45∼0.35); *P* < 0.05] and 45-min [SMD, −0.12 (95% CI, −0.38∼0.14); *P* < 0.05] gross movement interventions had detrimental effects on children’s physical fitness. However, there was a high level of heterogeneity in these studies (I^2^ > 85%). Sixty-minute gross movement interventions effectively improved children’s physical fitness [SMD, 0.47 (95% CI, 0.14–0.81); *P* < 0.05], and there was a moderate level of heterogeneity among these studies (I^2^ = 63.0%).

## 4 Discussion

This meta-analysis examined the influence of gross movement interventions on children’s physical fitness and explored the optimal intervention parameters (i.e., physical fitness factors, age groups, intervention duration, intervention frequencies and single intervention duration). The results show that gross movement interventions can significantly improve children’s physical fitness, with the strongest effects observed for explosive power. Additionally, we found that the effect of gross movement interventions was stronger for children aged 7–10 years, and the most effective intervention parameters included a frequency of once per week, a single-session duration of 60 min, and a total intervention duration of 16–18 weeks.

### 4.1 The influence of gross movement interventions on children’s physical fitness

In general, this study revealed that gross movement interventions can improve the physical fitness of children aged 3–10 years, which is consistent with previous research results (Niederer et al., 2012; Fang et al., 2017). Notably, there is a significant positive correlation between gross movement performance and physical fitness among children (Angilley and Haggas, 2009; Zhou, 2020), especially in terms of flexibility, explosive power and speed (Schott et al., 2007; Haga, 2009). As they age, children with high levels of gross movement ability exhibit greater physical fitness (Hands, 2008). This relationship suggests that gross movement interventions in early childhood will have a sustained positive impact on physical fitness, probably because children with strong gross motor ability will complete more complex physical activities, which may play a key role in the development of physical fitness.

Additionally, we found that gross movement interventions had the most significant effect on the explosive power of children aged 3–10 years, and no other studies reported such a significant effect (Hands, 2008; Wu et al., 2021; Chen and Gu, 2022). There are many reasons for thisresult. From the perspective of the experimental subjects included in the study, most of the subjects in this study were Chinese children, and explosive power changed significantly after the gross movement intervention, which may be related to genetic factors (Brandes, 2012). From the perspective of test methods, the experimental subjects included in the study mainly used standing long jumps to test children’s explosive power. A standing long jump is an important training method for improving explosive power, with medium and low intensity and low technical requirements (Dong et al., 2022). Furthermore, it is easier for children to master this skill. However, the excitability of the central nervous system is an important factor affecting explosive power (Wu et al., 2018; Chen et al., 2021), and running and jumping during gross movement interventions effectively improve the excitability of children’s nervous system (Loukia and Dimitris, 2022), thus recruiting more muscle fibers to participate in activities in the short term and improving the quality of explosive power. Therefore, among physical factors, gross movement interventions have the strongest effect on explosive power among children aged 3–10 years. However, it is necessary to consider the influence of genetic factors and testing methods.

### 4.2 The influence of gross movement interventions on the physical fitness of children of different ages

The effect of gross movement interventions on the physical fitness of children aged 7–10 years was greater than that on the physical fitness of children aged 3–6 years, which is consistent with some previous research results (Latorre Román et al., 2017; Li et al., 2019; Xin et al., 2019). The development and completion of gross movement is influenced by the integration of complex factors such as physiology, psychology, environment and task (Robinson et al., 2015; Li et al., 2019). This may reflect that as children age, they experience development in terms of their bones, muscles, cognitive abilities and nervous system, and they show increased adaptability to complex motor skills. Therefore, the effect of gross movement interventions on physical fitness is stronger among 7- to 10-year-old children. Previous studies have confirmed that children usually consolidate their learning and engage in continuous practice after the age of 6 (Venetsanou and Kambas, 2010; Chow and Louie, 2013), and mastery of gross motor skills begins to increase (Haywood and Gallahue, 2014). With increasing age, the mastery of gross motor skills significantly increases (Zhou, 2020). However, there are also studies showing that some physical fitness factors decrease with age (Gulías-González et al., 2014; Lopes et al., 2017; Godoy-Cumillaf et al., 2020), possibly related to the number of training sessions, the length of training sessions or other factors (Lopes et al., 2013).

### 4.3 Effects of different intervention durations on the physical fitness of children aged 3–10 years

When the intervention frequency was 10–12 weeks, the improvement effect of gross movement interventions on children’s physical fitness reached a significant level. The effect peaked when the intervention lasted 16–18 weeks. After 18 weeks, the effect of the intervention decreased slightly. This finding is consistent with the findings of a study that confirmed the positive impact of medium- to long-term gross movement interventions on physical fitness in children (Jaksic et al., 2020). This may be related to the improvement of motor skill levels under medium- and long-term interventions; in particular, after approximately 10 weeks of physical activity intervention, children’s motor skill levels are significantly improved, and the intervention benefits on children’s physical fitness persist (Bellows et al., 2013; Roth et al., 2015). It is also possible that improvements in children’s cognitive function have a positive impact on physical fitness. Gross movement is positively correlated with cognitive function (Thelen, 1995). A study of the effects of sports interventions on children’s cognitive function showed that after approximately 10 weeks of physical activity intervention, children’s cognitive function improved, and physical health benefits increased (Fisher et al., 2011). Another study from the perspective of child psychology and modern medicine supported the positive impact of children’s motor development on cognitive function (Wu et al., 2023). It can be inferred that improving children’s cognitive function through gross movement interventions can effectively enhance their physical fitness.

### 4.4 Effects of different intervention frequencies on children’s physical fitness

Gross movement intervention once a week had the most significant impact on the physical fitness of children aged 3–10 years, indicating that low-frequency interventions may be more helpful for improving children’s physical fitness, which is similar to the findings of some related studies (Granacher et al., 2016; Wu et al., 2021). Some studies have shown that intervention frequencies of 2–3 times per week (Latorre Román et al., 2017; Wu et al., 2023) and 4–5 times per week (Wang et al., 2021) have the greatest effects. However, the subjects of the interventions, the duration of the interventions and the durations of the single sessions were not the same in these studies. The frequency of intervention is affected by a variety of factors, but we must consider that the purpose of any intervention is not only to improve children’s physical fitness but also to motivate children to actively change unhealthy behaviors or habits (e.g., sitting for a long time) outside the intervention (Wang et al., 2021; Sheng et al., 2023), thus encouraging them to actively participate in physical activities. These findings need to be confirmed by more empirical studies.

### 4.5 Effects of different single-session durations on children’s physical fitness

Gross movement interventions lasting for 60 min per session had the greatest effect on the physical fitness of children aged 3–10 years, which is consistent with the results of most studies (Logan et al., 2012; Zhang et al., 2017; Shi, 2022; Shen and Dan, 2023). In 2022, China issued the Guidelines for Quality Assessment of Kindergarten Care Education, stating that children should engage in at least 60 min of physical activity per day (Ministry of Education, 2022). The results obtained in this study are consistent with the recommendations of those guidelines. However, some studies have shown that a single intervention duration of 30–40 min has the greatest effect (Xin et al., 2019) because children have short attention spans (Riethmuller et al., 2009). Overly long interventions are likely to result in children losing their focus (Logan et al., 2012). Overall, the current study still needs to further explore the critical value of the duration of a single intervention on physical activity. This may also account for the actual intervention process; for example, in an activity intervention, the actual gross movement practice time may only be two-thirds of the total time when accounting for warm-ups, recovery and rest (Brian et al., 2017). Additionally, the physiological and psychological characteristics of children should be considered in the design of intervention programs, focusing on gross movement interventions in different situations.

## 5 Research significance and future research

### 5.1 Theoretical significance

The decline in children’s physical fitness has become a global concern, engaging the attention of various sectors worldwide (Burns et al., 2018; Godoy-Cumillaf et al., 2021; Qu, 2021). This concern spans numerous countries, including China (National Health Monitoring Centre, 2022), Switzerland (Tomkinson, 2007), Russia (Malina and Katzmarzyk, 2006), Canada (Tremblay et al., 2010), and Spain (Moliner-Urdiales et al., 2010). The impact of children’s physical fitness levels on preventing and ameliorating conditions such as obesity and cardiovascular diseases is of paramount importance (Smith et al., 2014; Al-Mallah et al., 2018; Elagizi et al., 2018; Mintjens et al., 2018; Henriksson et al., 2020). However, recent research has predominantly focused on identifying correlations between different gross movements and children’s physical fitness (Foulkes et al., 2021; Liu et al., 2023) or differential performance (Haugen and Johansen, 2018; Zheng et al., 2022). There is a notable gap in the exploration of the maximum effect sizes of gross movement interventions in enhancing children’s fitness. Acknowledging the pressing need for the development of children’s physical fitness, this study thoroughly investigated the optimal effect sizes of gross movement interventions for children aged 3–10 years from various perspectives. These include physical fitness components, age groups, intervention duration, intervention frequency, and single-session duration. This study provides a theoretical reference for the formulation of interventions to enhance children’s physical fitness. Moreover, this research leverages findings from diverse fields, such as health, sports, and education, as primary data. By adopting an interdisciplinary approach encompassing kinesiology and education, this study involved a systematic review to strategically address contemporary issues that scholars are currently focusing on, providing theoretical guidance for related fields of study.

### 5.2 Practical significance

Practical evidence attests to the effectiveness of gross movement interventions in enhancing children’s physical fitness, addressing health concerns such as childhood obesity and cardiovascular issues (Okely et al., 2004; Barnett et al., 2008; Robinson et al., 2015; Ma and Song, 2017; Pu and Yang, 2017; Hao, 2018; Li et al., 2019; Wang et al., 2019). However, the optimal organization of gross movement interventions for enhancing children’s physical fitness remains a pivotal area of inquiry. Questions surrounding which age group benefits most from the impact of gross movement interventions on physical fitness and which specific physical fitness components show the most significant improvement necessitate thorough exploration. In light of these considerations, the findings of this study offer scientific intervention guidance for enhancing children’s physical fitness, offering benefits to society, educational institutions, and families. First, during childhood, emphasis should be placed on practicing gross movements, given that they have a significant impact on improving children’s physical fitness, and this positive correlation remains as children grow older. Second, the cultivation of explosive strength in children is of paramount importance. Focus on enhancing children’s explosive strength through tailored gross movement interventions, including movements such as tuck jumps and competitive rope climbing, can effectively enhance their explosive strength. Third, the age bracket of 7–10 years emerges as the period when children experience the most rapid improvement in physical fitness, although attention should be given to factors such as training frequency and duration. Fourth, a gross movement intervention with a 16- to 18-week duration has been shown to be effective in enhancing children’s physical fitness, contributing to the refinement of their skills and cognitive abilities. Fifth, recognizing the impact of unhealthy habits or behaviors on children’s physical fitness is crucial, and a weekly session of gross movements is deemed optimal for the most pronounced effects on enhancing children’s physical fitness. Finally, judiciously arranging the session duration to be 60 min per session is shown to be optimal for improving children’s physical fitness. In summary, the findings of this study provide critical practical references for effectively enhancing children’s physical fitness. Societal, educational, and familial entities should prioritize the influential role of gross movements in improving children’s physical fitness and implement effective measures to prevent childhood physical illnesses, ultimately reducing the global incidence of developmental disorders in children.

### 5.3 Future research

In a follow-up study, this research group will further explore the following: ① the ongoing impact of gross movement interventions on children’s physical fitness; ② the effects of different gross movement interventions on different physical qualities; and ③ the effect of different total intervention durations on children’s physical fitness and related issues.

## 6 Limitations

This study did not discuss the subsequent effects of gross movement interventions on children’s physical fitness. In addition, gross movements are mainly completed using large muscle groups. This study did not classify gross movements by muscle groups, nor did it explore the effects of different large muscle groups on physical fitness. Second, the literature included in this study did not account for the effects of sex, country, total duration of intervention across age groups, family environment, nutrition and health status, teacher training level or other factors related to on children’s physical fitness, which may have led to certain biases in the results. Therefore, additional studies are needed to overcome these limitations and validate the current findings.

## 7 Conclusion

The results of this meta-analysis show that gross movement interventions have a positive impact on the physical fitness of children aged 3–10 years, especially on explosive power. Specifically, gross movement interventions had stronger effects on the physical fitness of children aged 9–10 years. Additionally, the optimal intervention parameters include one intervention per week, 60 min per session, and a total intervention duration of 16–18 weeks.

## Data availability statement

The original contributions presented in this study are included in the article/supplementary material, further inquiries can be directed to the corresponding author.

## Author contributions

LH: Writing – review and editing, Writing – original draft, Visualization, Resources, Methodology, Investigation, Funding acquisition, Data curation. SW: Writing – original draft, Project administration, Methodology, Investigation, Funding acquisition, Writing – review and editing. QL: Formal analysis, Conceptualization, Writing – review and editing. GN: Writing – review and editing, Formal analysis, Investigation, Funding acquisition.
